# Light‐controlled scaffold‐ and serum‐free hard palatal‐derived mesenchymal stem cell aggregates for bone regeneration

**DOI:** 10.1002/btm2.10334

**Published:** 2022-05-13

**Authors:** Zhiwei Jiang, Na Li, Qin Shao, Danji Zhu, Yuting Feng, Yang Wang, Mengjia Yu, Lingfei Ren, Qianming Chen, Guoli Yang

**Affiliations:** ^1^ Stomatology Hospital, School of Stomatology Zhejiang University School of Medicine Hangzhou Zhejiang China; ^2^ Zhejiang Provincial Clinical Research Center for Oral Diseases Hangzhou Zhejiang China; ^3^ Key Laboratory of Oral Biomedical Research of Zhejiang Province Cancer Center of Zhejiang University Hangzhou Zhejiang China

**Keywords:** bone regeneration, cell aggregates, hard palate, light‐controlled, mesenchymal stem cells

## Abstract

Cell aggregates that mimic in vivo cell–cell interactions are promising and powerful tools for tissue engineering. This study isolated a new, easily obtained, population of mesenchymal stem cells (MSCs) from rat hard palates named hard palatal‐derived mesenchymal stem cells (PMSCs). The PMSCs were positive for CD90, CD44, and CD29 and negative for CD34, CD45, and CD146. They exhibited clonogenicity, self‐renewal, migration, and multipotent differentiation capacities. Furthermore, this study fabricated scaffold‐free 3D aggregates using light‐controlled cell sheet technology and a serum‐free method. PMSC aggregates were successfully constructed with good viability. Transplantation of the PMSC aggregates and the PMSC aggregate‐implant complexes significantly enhanced bone formation and implant osseointegration in vivo, respectively. This new cell resource is easy to obtain and provides an alternative strategy for tissue engineering and regenerative medicine.

## 1 INTRODUCTION

Mesenchymal stem cells (MSCs) have been applied in bone regeneration,^1^, ^2^ bone‐tendon healing,^3^ cartilage formation,^4^ and dental pulp regeneration.^5^ MSCs usually originate from bone marrow. However, these stem cells are donated by other patients and they may show immunogenicity. Therefore, autologous cells are preferred in the clinic. Hard palates may provide a new, promising seeding cell choice. Hard palate tissues have a strong regenerative ability. After surgical procedure, the wound can heal rapidly without scarring. Many studies have proven that MSCs derived from oral cavity can promote not only dental tissue regeneration, including dental pulp,^6^ periodontal tissue,^7^ and dentin,^8^ but also bone^9^ and nerve.^10^ These MSC resources included dental pulp,^11^ gingiva,^12^ periodontal ligament,^13^ exfoliated deciduous teeth,^14^ apical papilla,^15^ dental follicle,^16^ hard palatal adipose tissue,^17^ and hard palatal periosteum,^18^ and so on. However, to the best of our knowledge, no studies have reported MSCs derived from a rat hard palate (palatal‐derived mesenchymal stem cells [PMSCs]).

MSCs are vital cell resources for fabrication of cell aggregates. Cell aggregates are widely used in a range of fields, including tissue engineering,^19^ drug testing,^20^ and cancer research.^21^ Recently, cell aggregates have been constructed with microparticles,^22^ magnetic nanoparticles,^23^ and hydrogels.^19^ However, these methods would incorporate foreign materials that might harm cell viability. Therefore, there is a pressing need to develop cell aggregates without foreign harmful materials. Previous studies have reported that light‐controlled cell sheet technology combined with vitamin C could fabricate cell sheets in a convenient and safe manner.^24^, ^25^, ^26^ Furthermore, light is an easy‐to‐control source, saving time and increasing efficiency during experiments.^27^ However, few investigations have reported the successful harvest of cell aggregates under light illumination. In this study, we used a light‐controlled method to fabricate cell aggregates.

Cell aggregates can mimic native cellular microenvironments in vivo.^28^ In particular, MSCs isolated from hard palates are promising cell sources for in vivo transplantation owing to their advantages of easy isolation, multipotent differentiation, and fast proliferation. Cell–cell/cell‐extracellular matrix (ECM) interactions have improved cell viability compared with single cells.^29^ Cell aggregates could limit the immobility of cells at the site of the defect and improve transplantation efficiency.^30^ Moreover, they have better osteogenic differentiation potential than cell sheets.^31^ These factors may contribute to the improved bone healing ability of cell aggregates. Therefore, we assumed that PMSC aggregates could accelerate bone regeneration in vivo.

The composition of serum is complex and has not been completely determined. It contains a large number of microorganisms that may have adverse effects on cell growth.^32^ Moreover, the storage life of serum is limited, and there is high variability between batches.^33^ Therefore, it is not ideal for clinical applications. Recently, it was found that MSCs could form cell aggregates in serum‐free culture medium.^34^, ^35^ This procedure had several advantages, such as a low probability of microbiological contamination or transmission of animal diseases to humans, low cost, and high reproducibility.^23^, ^36^ Cell sheet technology combined with serum‐free culture may provide a novel method for reliable clinical applications of cell aggregates to guarantee well‐defined compositions with a low risk of contamination.

The aims of this study were to isolate rat PMSCs and evaluate the feasibility of harvesting scaffold‐free and serum‐free cell aggregates via light‐controlled cell sheet technology. Furthermore, we aimed to evaluate the bone regeneration capacity of PMSC aggregates.

## 2 RESULTS

### 2.1 Harvesting procedure and healing process of PMSCs

**FIGURE 1:**
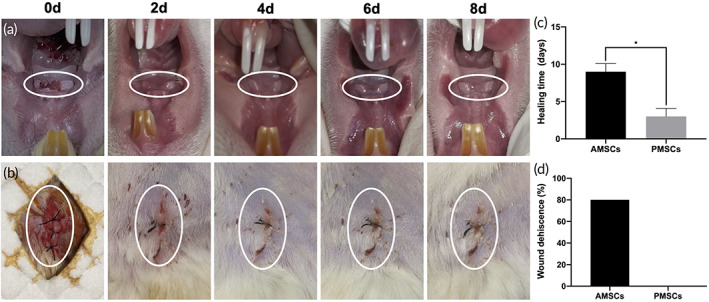
Wound healing processes of palatal‐derived mesenchymal stem cells (PMSCs) and adipose‐derived mesenchymal stem cells (AMSCs) harvesting. (a) A long strip of mucosa of 1.5 mm × 3 mm was removed from the hard palate. The wound showed a pink, healthy healing appearance without infection from the second day after surgery. (b) Subcutaneous adipose tissue was removed from the inguinal region and the harvesting wound was intermittently sutured. The healing time of the wound was approximately 8 days. During this period, the wound was prone to dehiscence. (c) The healing time of PMSCs harvesting was significantly shorter than that of AMSCs. (d) All the hard palatal mucosa wounds were healed without incident, while the adipose tissue harvesting sites had a high wound dehiscence rate. *: *p* <0.05

Oral mucosa was harvested from the rat hard palate and the wounds were left to heal naturally without sutures. To evaluate the healing process of PMSCs harvesting, we closely observed and recorded the wound appearance after surgery (Figure 1a). After isolations of PMSCs and adipose‐derived mesenchymal stem cells (AMSCs; Figure 1b), the wound healing time in the PMSCs group (3 ± 1.10 days) was significantly shorter than that in the AMSCs group (9 ± 1.10 days; Figure 1c). Furthermore, the PMSCs group had no risk of wound dehiscence, while 80% of rats had wound dehiscence in the AMSCs group (Figure 1d).

### 2.2 Characteristics of the PMSCs

**FIGURE 2:**
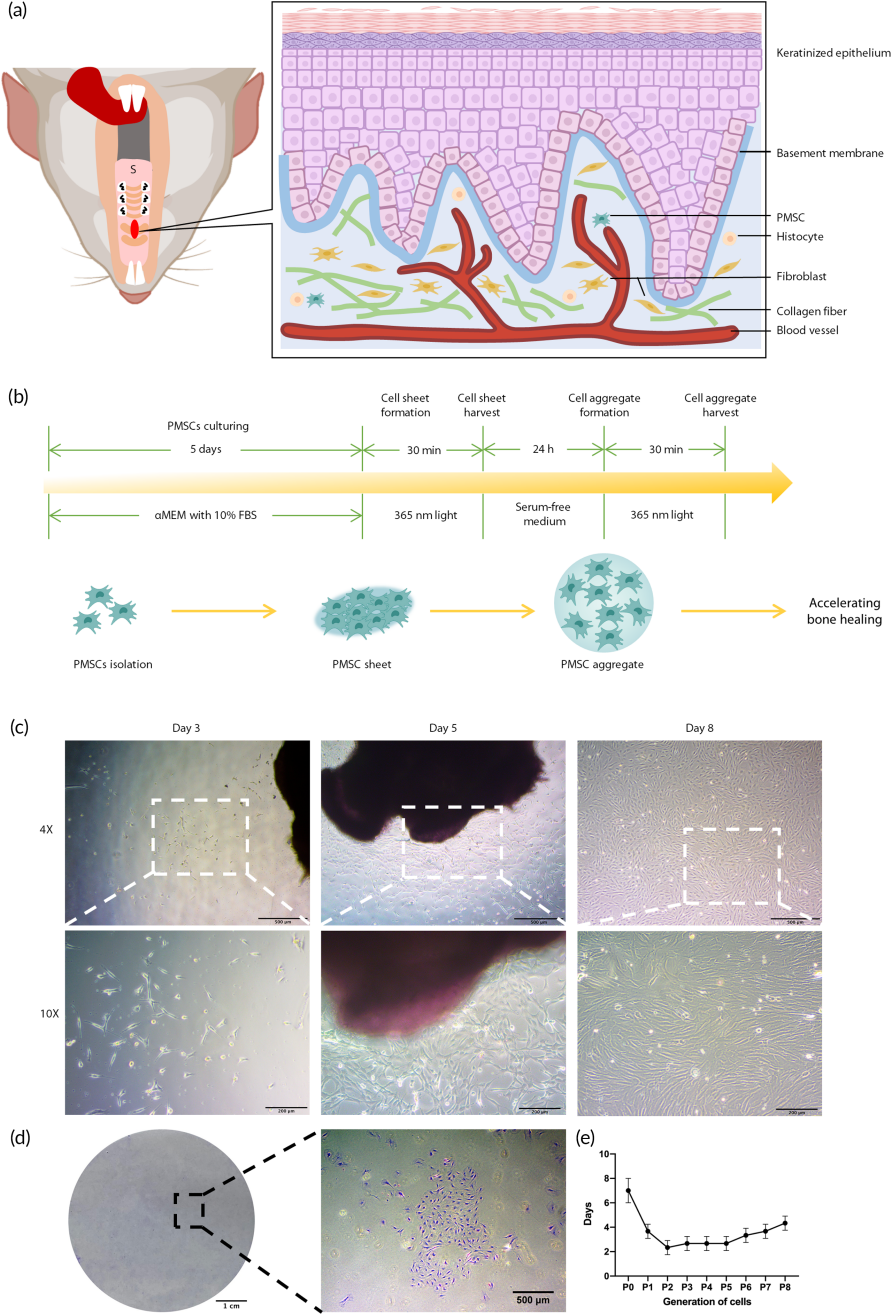
Isolation, culture, and characteristics of palatal‐derived mesenchymal stem cells (PMSCs). (a) Left: Schematic representation of the rat hard palate (intraoral view). The red oval indicates the location of the sampling position. Right: Histological pattern diagram of the rat hard palate. The hard palate mucosa consisted of a keratinized epithelium layer and a lamina propria layer. PMSCs located near the basement membrane between the latter two layers. (b) The technical protocol of this study. We isolated and cultured PMSCs, and then harvested PMSC sheets using light‐activated cell sheet technology. After transferring PMSC sheets into serum‐free medium, PMSC aggregates formed and were collected under light illumination. (c) Isolated PMSCs gradually migrated from the tissues in culturing medium on day 3, 5, and 8. (d) Cell colonies formed. (e) The culture time decreased between passage 0 and passage 3 and then increased. Scale bars: (c) first row: 500 μm, second row: 200 μm, (d) first figure: 1 cm, second figure: 500 μm

PMSCs originated from the lamina propria layer of the hard palate and were close to the basement membrane (Figure 2a). In this study, we isolated PMSCs and turned them into cell sheets and aggregates, which were easily and safely harvested under light activation (Figure 2b). As Figure 2c shows, spindle‐shaped cells migrated from the hard palate tissue, congregated and finally formed cell colonies (Figure 2d). In addition, the culturing time decreased from passage 0 to passage 3, while after passage 3, the culture time gradually increased (Figure 2e).

**FIGURE 3:**
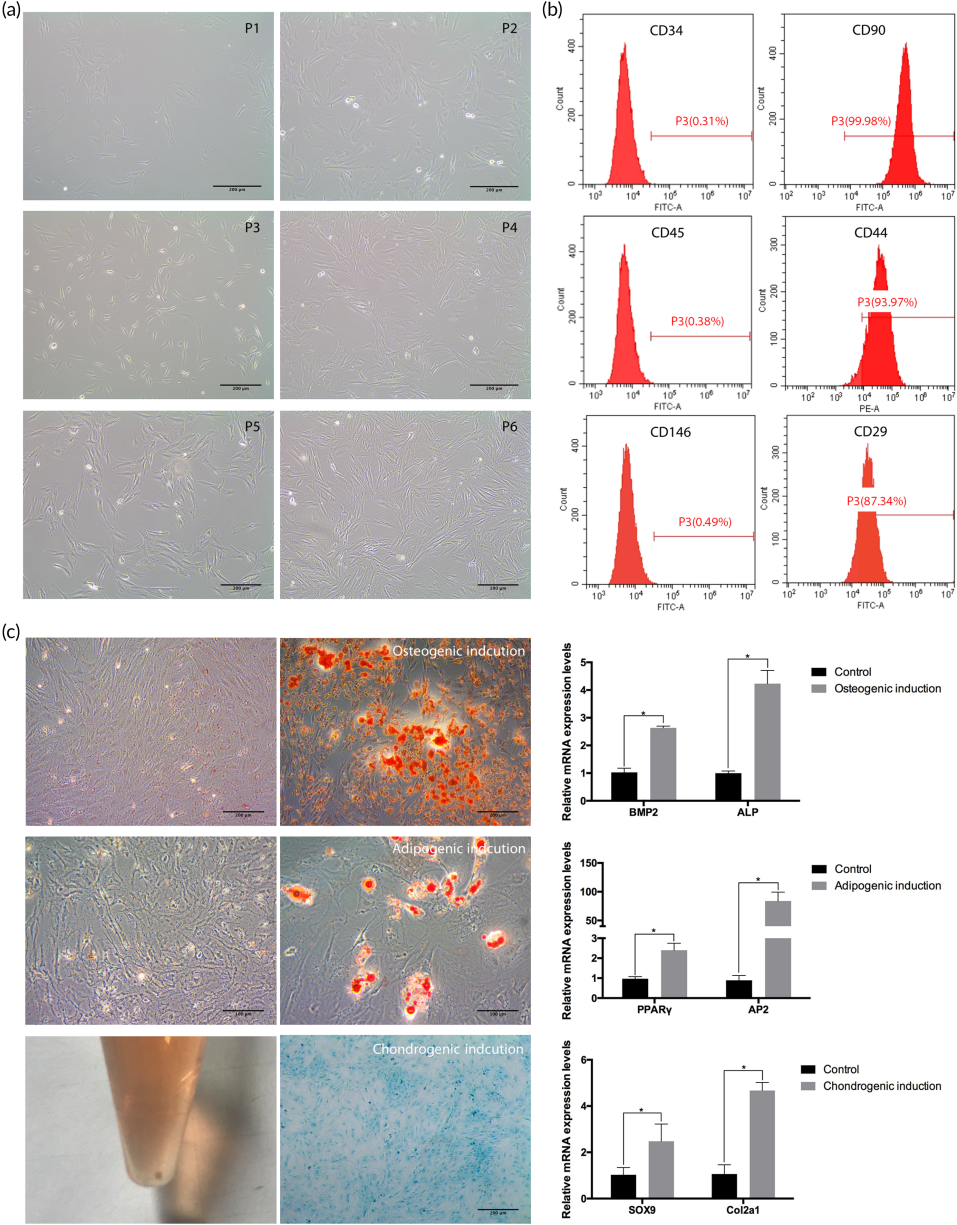
Identification of palatal‐derived mesenchymal stem cells (PMSCs). (a) PMSCs from passage 1 (P1) to passage 6 (P6) were spindle‐shaped. (b) Flow cytometry analysis showed that PMSCs were positive for CD90, CD44, and CD29, and negative for CD34, CD45, and CD146. (c) The staining was positive after osteogenic, adipogenic, and chondrogenic differentiation. In the meantime, the osteogenic (BMP2, ALP), adipogenic (PPARγ, AP2), and chondrogenic (SOX9, Col2a1) gene expression levels were significantly elevated. *: *p* <0.05. Scale bar: (a) 200 μm, (c) first and third row: 200 μm, second row: 100 μm

PMSCs remained spindle‐shaped from passage 1 to passage 6 (Figure 3a). Results of flow cytometry indicated that the PMSCs were positive for CD90, CD44, and CD29 and negative for CD34, CD45, and CD146 (Figure 3b). After culture in osteogenic, adipogenic, and chondrogenic medium, the PMSCs were positive for alizarin red staining, oil red O staining, and alcian blue staining. RT‐qPCR assays showed that the expression levels of osteogenic (bone morphogenetic protein‐2 [BMP2] and alkaline phosphatase [ALP]), adipogenic (peroxisome proliferator‐activated receptor γ [PPARγ] and adipocyte protein 2 [AP2]), and chondrogenic (SRY‐related high mobility group‐box gene 9 [SOX9] and collagen type II alpha 1 [Col2a1]) genes were significantly enhanced (*p* <0.05, Figure 3c).

**FIGURE 4:**
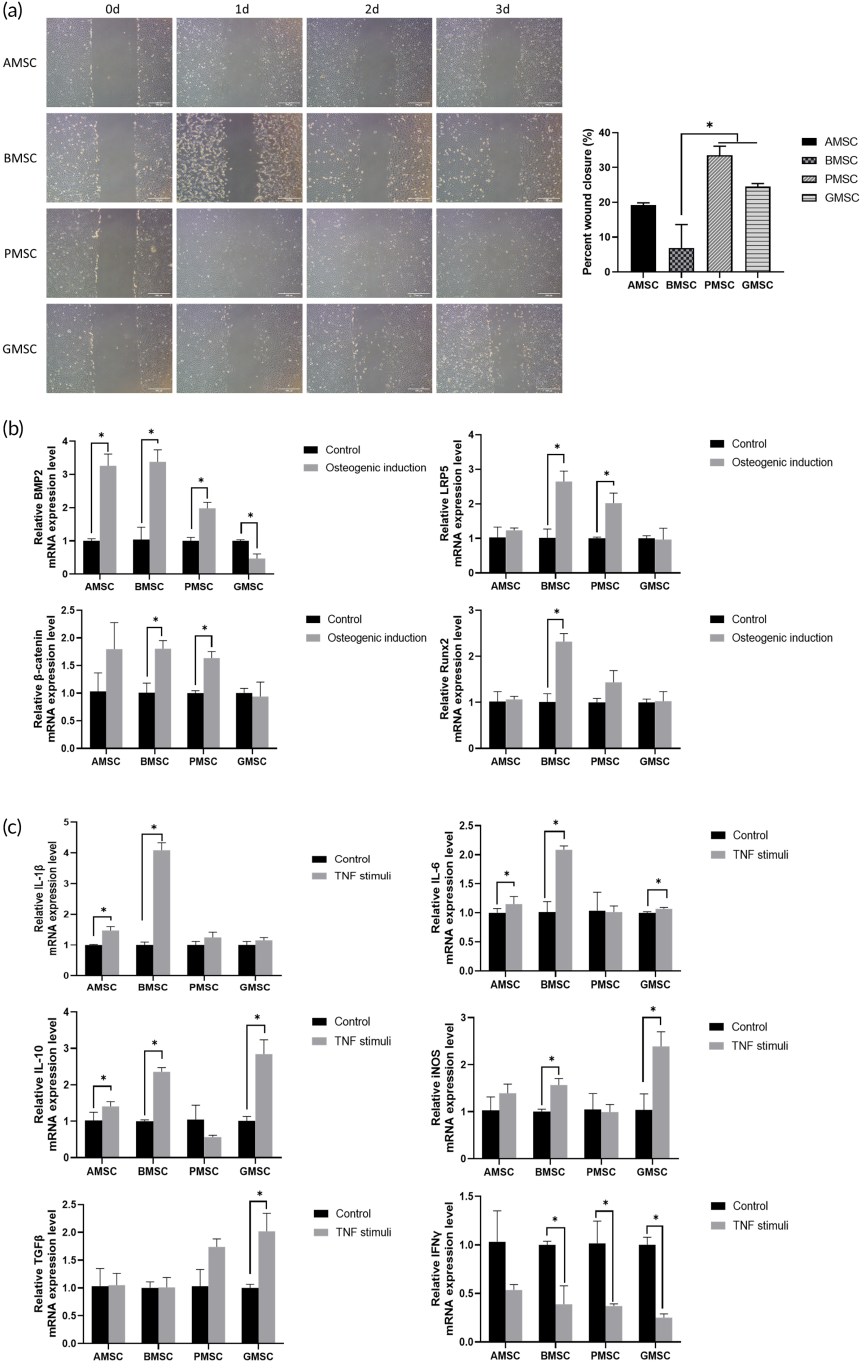
Comparisons of characteristics of palatal‐derived mesenchymal stem cells (PMSCs) and other cell sources. (a) Exemplary images of four kinds of cells of wound closure. Results showed that percent wound closure was significantly higher in PMSC and gingival‐derived mesenchymal stem cell (GMSC) groups compared to bone mesenchymal stem cell (BMSC) group. (b) The osteogenic gene expression level changes in four kinds of cells. PMSCs exhibited relatively positive reaction to osteogenic induction. (c) The inflammation‐related gene expression level changes after TNF‐α stimulation. Pro‐inflammatory gene expression levels were significantly elevated. *: *p* <0.05. Scale bar: (a) 200 μm

To learn more about the characteristics of PMSCs, several experiments were performed, comparing PMSCs with other three kinds of cells. Cell scratch assays showed that the two cell sources derived from oral mucosa, namely PMSCs and gingival‐derived mesenchymal stem cells (GMSCs), had similar high migration ability (Figure 4a). The wound closure rates were 24.5 ± 0.8% and 21.0 ± 3.3%, respectively. On the contrary, bone mesenchymal stem cells (BMSCs) exhibited a significantly lower migration ability with a wound closure rate of 6.9 ± 6.7% (*p* <0.05). To evaluate the cellular responses under different circumstances, cells were subjected to osteogenic induction and tumor necrosis factor‐α (TNF‐α) stimulation. RT‐qPCR assays showed that the expression levels of osteogenic (BMP2, low‐density lipoprotein receptor‐related protein 5 [LRP5], and β‐catenin) were significantly increased in PMSCs (Figure 4b). BMSCs exerted the most positive response to osteogenic induction with high levels of related gene expression. On the contrary, GMSCs were not able to be induced by osteogenic medium. Finally, the expression levels of genes associated with inflammation were estimated (Figure 4c). AMSCs, BMSCs, and GMSCs showed noticeable changes in the mRNA expression of interleukin‐1β (IL‐1β), interleukin‐6 (IL‐6), interleukin‐10 (IL‐10), or inducible nitric oxide synthase (iNOS), while PMSCs seemed to be more insensitive to inflammatory induction. GMSC group showed significant increase in the expression of transforming growth factor‐β (TGFβ). Except for AMSCs, all cell sources showed remarkable decrease of interferon γ (IFNγ).

### 2.3 Characteristics of the PMSC sheets and PMSC aggregates

**FIGURE 5:**
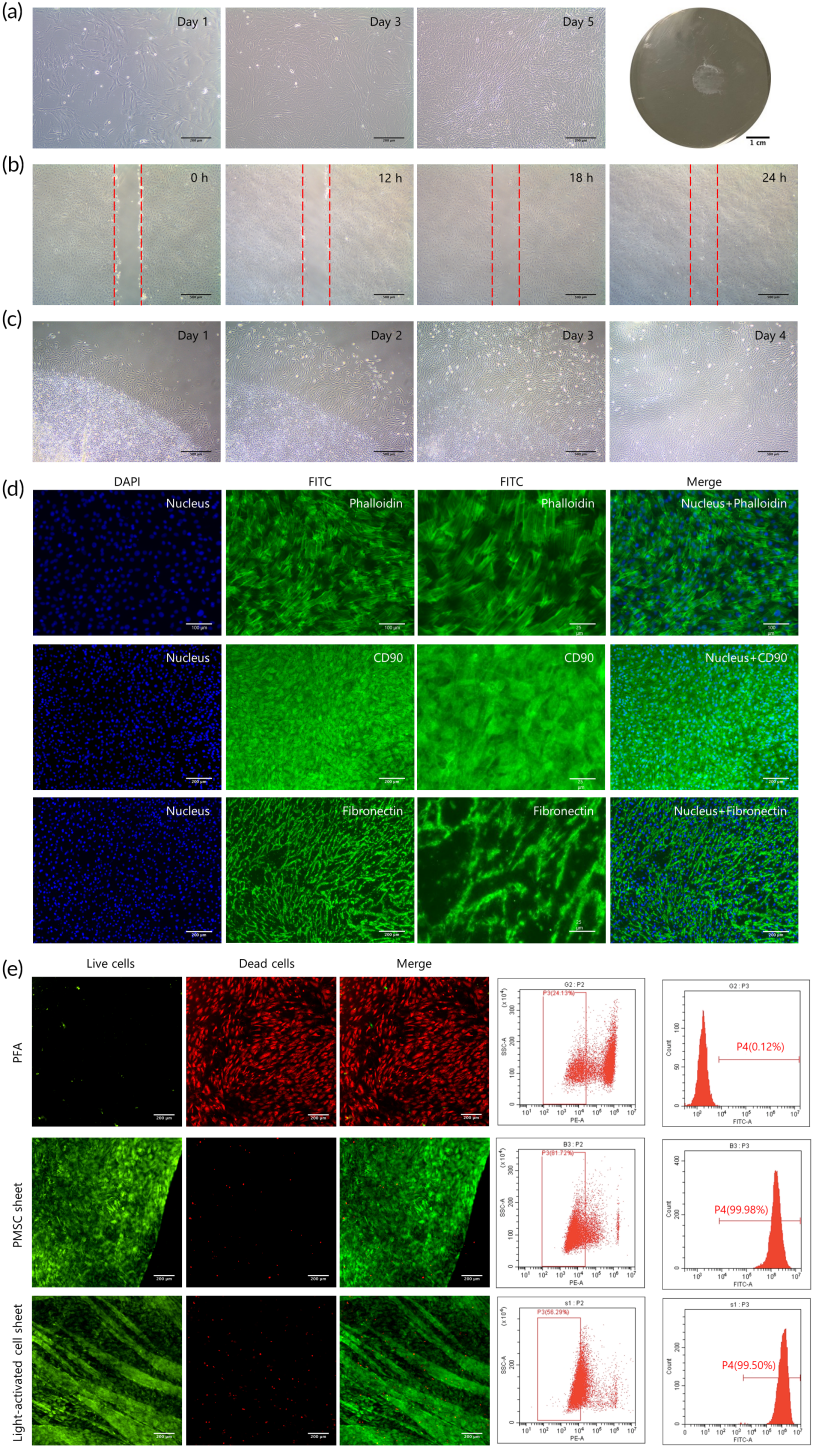
Characteristics of palatal‐derived mesenchymal stem cell (PMSC) sheets. (a) PMSCs proliferated on the nanodot platforms from day 1 to 5, and finally formed cell sheets. (b) PMSC sheets could heal rapidly in 24 h after injury. (c) The harvested PMSC sheets could reattach onto plates. (d) Large amounts of cells and fibronectin could be observed in the PMSC sheets. PMSC sheets were positive for CD90. (e) Few cells (0.12%) lived after immersion in PFA, while most cells in the PMSC sheets and light‐activated cell sheets survived (99.98% and 99.50%, respectively). Scale bars: (a) first three figures: 200 μm, last figure: 1 cm, (b,c) 500 μm, (d) first, second, and fourth lines: 100 and 200 μm, third line: 25 μm, (e) 200 μm

After culture on nanodot platforms for 5 days, PMSCs proliferated and formed intact cell sheets (Figure 5a). The migrating and reattaching capacities of the PMSC sheets were examined. As Figure 5b shows, they were able to heal within 24 h after injury. The harvesting procedure under 365 nm light illumination did not harm the reattachment of the PMSC sheets (Figure 5c).

The immunofluorescence results showed that the PMSC sheets were abundant in cells and ECM, with a large amount of fibronectin. As a complement to the flow cytometry analysis, PMSC sheets were observed to be positive for CD90, one of the MSC markers, under an immunofluorescence microscope (Figure 5d). The live‐dead staining results of the PMSC sheets confirmed their viability (Figure 5e). Few live cells (0.12%) remained after immersion in paraformaldehyde (PFA), which was the negative control, while most cells in the adherent and detached PMSC sheets after light activation exhibited good viability (99.98% and 99.50%, respectively), demonstrating the safety of the culturing and harvesting process.

**FIGURE 6:**
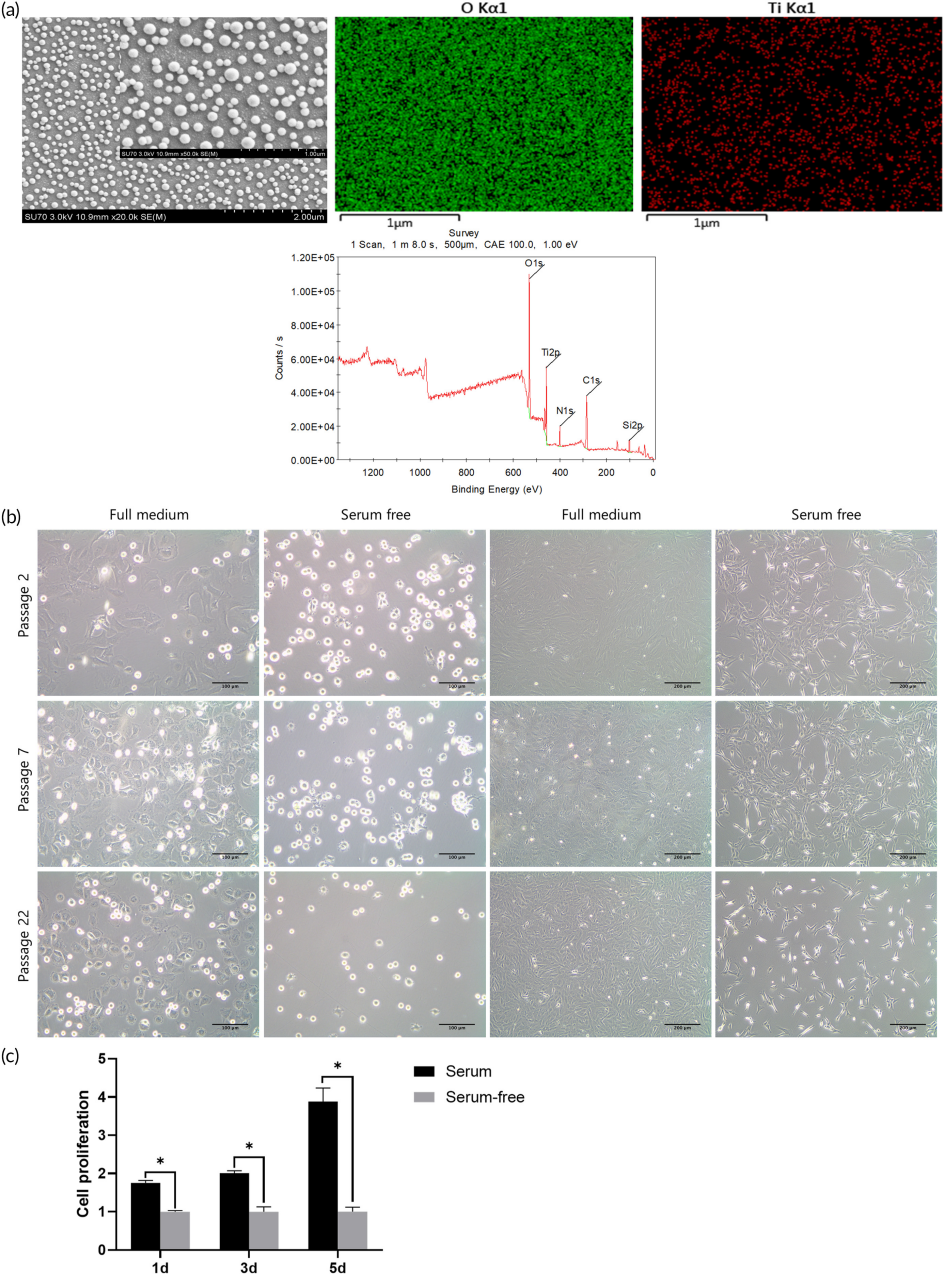
Fabrication of palatal‐derived mesenchymal stem cell (PMSC) aggregates using light‐controlled TiO_2_ nanodot platform and a serum‐free method. (a) The scanning electron microscopy (SEM) image showed that nanodots were evenly distributed on the TiO_2_ platforms. The energy‐dispersive X‐ray spectroscopy (EDS) results showed that O and Ti are two major elements on the TiO_2_ nanodot platforms. (b) Morphology of the PMSC sheets of passage 2 (P2), passage 7 (P7), and passage 22 (P22) cultured in complete medium and serum‐free medium. (c) Evaluation of the cell growth rates of PMSCs cultured with α‐MEM containing 10% FBS and serum‐free medium. Scale bars: (a) first figure: 1 and 2 μm, second and third figures: 1 μm, (b) first and second lines: 100 μm, third and fourth lines: 200 μm

The TiO_2_ nanodot platforms were observed using scanning electron microscopy (SEM). O, C, N, Ti, and Si were detected, among which O and Ti were the major elements on the surfaces (Figure 6a), indicating that the culturing platform was clean and uncontaminated. The culturing procedure from PMSCs to cell aggregates is summarized in Figure 2b. Both cell sheets and cell aggregates were harvested safely from the TiO_2_ nanodot platform under 365 nm light illumination, while the culturing environment was different. Common alpha‐modified minimum essential medium (α‐MEM) with 10% fetal bovine serum (FBS) was used to form cell sheets, while serum‐free medium was used for cell aggregate generation. PMSCs were spindle‐shaped in complete medium, which was beneficial for their adhesion. When transferred into serum‐free medium, the cells tended to become round and separate, possibly making it easier to self‐assemble and turn into cell aggregates (Figure 6b). According to the results of Alamar Blue assay (Figure 6c), the cell growth rate was lower in serum‐free culture medium. The difference of proliferation rate between two groups of PMSCs was increasingly evident with the passage of culture time. UV light illumination at 365 nm induced cell sheet detachment, and serum‐free medium turned the cell sheets into cell aggregates. After an additional irradiation treatment, the cell aggregate was harvested from the TiO_2_ nanodot platform.

**FIGURE 7:**
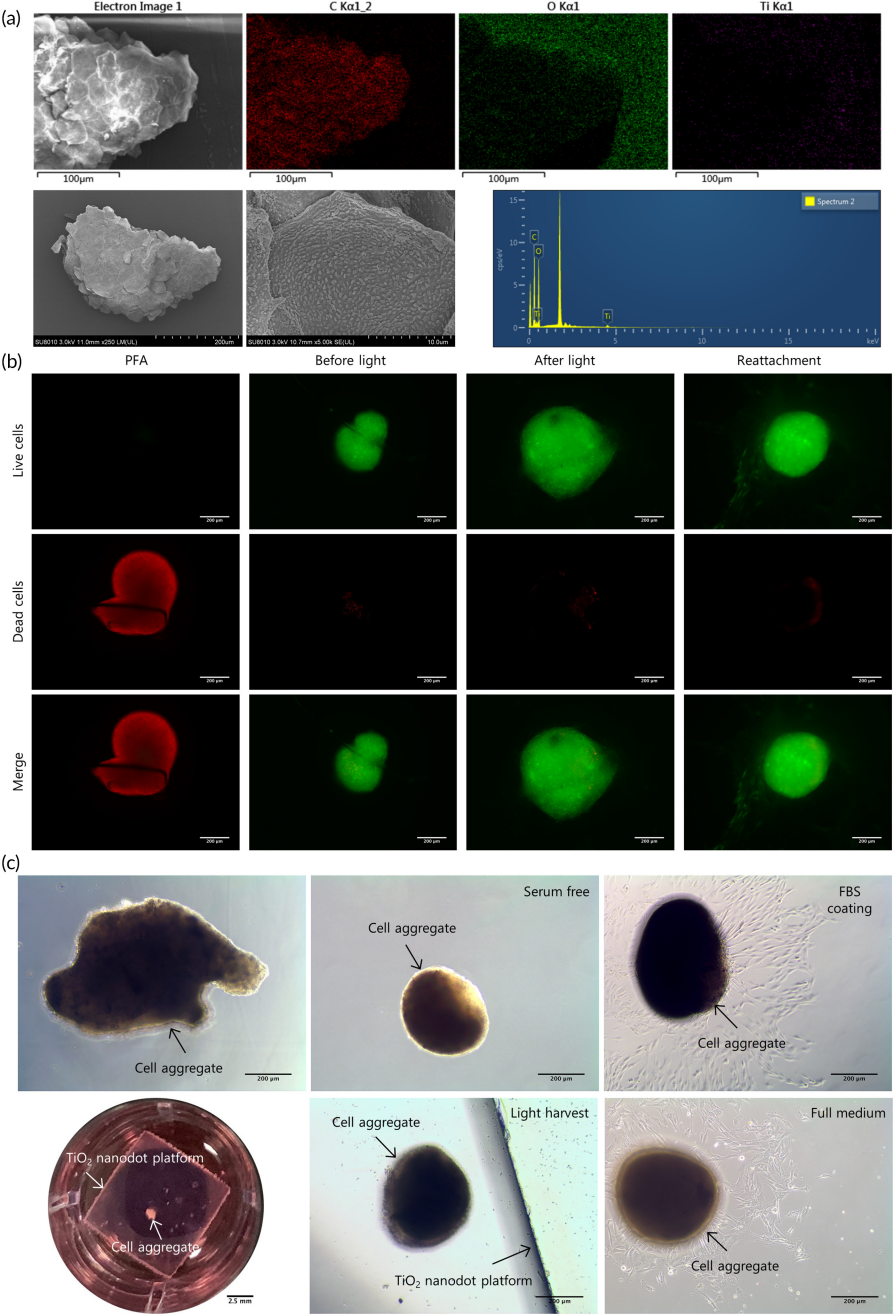
Characteristics of palatal‐derived mesenchymal stem cell (PMSC) aggregates. (a) The energy‐dispersive X‐ray spectroscopy (EDS) analysis indicated that C, O, and Ti could be detected in the cell aggregates. The scanning electron microscopy (SEM) images showed that cell aggregate was composed of cells and rich extracellular matrix (ECM). (b) Live‐dead staining of the PMSCs at each step. The cells lost viability in PFA (first line), while the live cells had good viability before illumination (second line), after light illumination (third line) and after reattachment (fourth line). (c) Cell sheets on TiO_2_ nanodot platform transferred into cell aggregates after culture in serum‐free medium. When coated with FBS or placed back into complete medium, spindle‐shaped cells migrated from cell aggregates. Scale bar: (a) first row: 100 μm, second row: 200 and 10 μm, (b) 200 μm, (c) first row: 200 μm

Energy‐dispersive X‐ray spectroscopy (EDS) analysis implied the elemental composition of cell aggregates, and the SEM images depicted the detailed morphology, which was composed of regularly arranged cells and sufficient ECM (Figure 7a). Furthermore, the amplified images showed the healthy status of PMSCs before and after light illumination, as well as their capacity for reattachment (Figure 7b). Notably, when transferred back into medium with FBS, the spindle‐shaped cells migrated from the cell aggregates and then adhered to the platform (Figure 7c).

### 2.4 Biological evaluation of the PMSC aggregates in vivo

**FIGURE 8:**
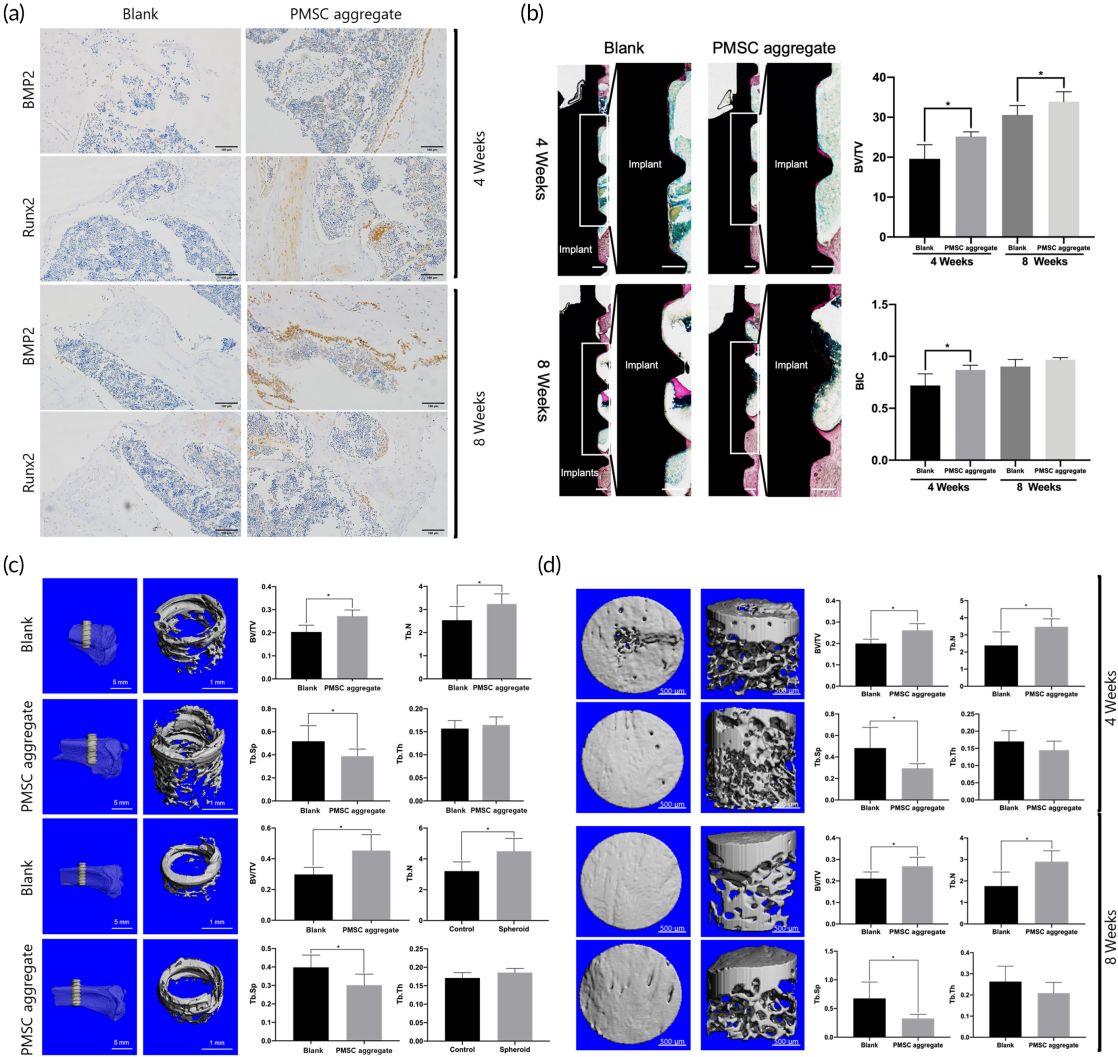
Bone regeneration evaluation of palatal‐derived mesenchymal stem cell (PMSC) aggregates in vivo. (a) Immunohistochemistry images of tibia defect healing with blank SLA implants and the PMSC aggregates‐implant complexes after 4 and 8 weeks. The expressions of BMP2 and Runx2 were significantly elevated in the PMSC aggregate group at the two healing points. (b) Hard tissue sectioning images of tibia defect healing with blank SLA implants and the PMSC aggregates‐implant complexes after 4 and 8 weeks. Bone volume/tissue volume (BV/TV) and bone‐implant contact (BIC) were measured. 3D bone regeneration evaluation in a tibial implant model (c) and a tibial defect model. (c) showed 3D images of tibia defect healing with blank SLA implants and the PMSC aggregates‐implant complex after 4 and 8 weeks, while (d) showed images of tibia defect healing without and with PMSC aggregates after 4 and 8 weeks. Multiple‐comparison analysis of BV/TV, Tb.N, Th.Sp, and trabecular thickness (Tb.Th) were performed. The PMSC aggregates significantly promoted bone regeneration. BV/TV and Tb.N were significantly elevated, while Tb.Sp was significantly decreased in the PMSC aggregate groups. *: *p* <0.05. Scale bars: (a) 100 μm, (b) 250 μm, (c) 5 and 1 mm, (d) 500 μm

Compared with large‐grit‐sandblasted and acid‐etched (SLA) implants, PMSC aggregates‐implant complexes were able to promote osseointegration. Immunohistochemistry images showed that BMP2 and Runx2 expressions were significantly higher in new bone around PMSC aggregate‐implant complexes compared with SLA implants after 4 and 8 weeks after implant insertion (Figure 8a). Additionally, histological examination for hard tissue sections showed that the PMSC aggregate group had higher bone‐implant contact (BIC) and bone volume/tissue volume (BV/TV; Figure 8b). Micro‐computed tomography (micro‐CT) showed that the tibias inserted with PMSC aggregate‐implant complexes had significantly higher BV/TV and trabecular number (Tb.N) values and a lower trabecular space (Tb.Sp) value than the tibias with the SLA implant (*p* <0.05, Figure 8c).

In the rat tibial defect model, micro‐CT analysis indicated that after 4 and 8 weeks of healing, BV/TV and Tb.N of the tibias with the PMSC aggregates were significantly higher than those of the control group, and Tb.Sp was significantly lower. As seen in the 3D reconstruction images, the injured tibias were much stronger and more intact in the PMSC aggregate group, representing a more satisfying healing outcome (Figure 8d).

## 3 DISCUSSION

Exploring a new resource of MSCs with advantages of easy isolation and rapid healing is a significant research issue in tissue engineering and regenerative medicine. Oral mucosa might be a simple and alternative MSCs resource due to its strong regenerative capacity.^37^, ^38^ Recently, the potential role of MSCs in enhancing bone formation in the clinics has been proven.^39^ In this study, we reported the isolation and culture of rat PMSCs with rapid wound healing and no risk of wound dehiscence for the first time. We investigated the fabricating strategy of PMSC aggregates through light‐controlled and serum‐free method, and evaluated their bone regeneration ability in vivo.

Our study is consistent with the previous studies that MSCs have played a leading role in regenerative medicine due to their self‐renewal ability and potential to differentiate into various cell types.^40^, ^41^, ^42^, ^43^ To the best of our knowledge, this is the first study to show MSCs could be isolated from postnatal rat hard palates. Previous studies have reported embryo palatal MSCs,^44^, ^45^, ^46^ palatal periosteum MSCs,^18^ and MSCs from adipose tissue of the hard palate.^17^ The oral mucosa, including the hard palate mucosa, is a rapidly dividing tissue with great regeneration capacity.^47^, ^48^ Compared to MSCs derived from other organs, such as bone marrow MSCs, PMSCs showed advantages with easier and less invasive harvesting procedures. Therefore, they may present great potential in future clinical applications. Trilineage differentiation analysis, proliferative capacity, and cell markers confirmed isolation of MSCs,^49^, ^50^, ^51^, ^52^ strongly supporting that PMSCs represent a MSC population. By comparing with traditional cell sources including AMSCs, BMSCs and GMSCs, we provided an initial impression of PMSCs. Cell scratch assays indicated excellent cell migration ability of these oral mucosa‐derived cells. This can be an advantage as cell migration is essential for various biological functions.^53^ Considering the potential application in osseointegration acceleration and bone defect healing, PMSCs were evaluated for its osteogenic capacity and tolerance to inflammation. In consistence with the results of trilineage differentiation assay, PMSCs showed positive response to osteogenic induction despite the degree of increase in osteogenic genes was relatively lower than BMSCs. As for TNF‐α stimulation, though the mRNA expression trends of pro‐inflammatory and anti‐inflammatory factors varied among the four kinds of cells, we might suppose that PMSCs were relatively insensitive to inflammatory challenge. This is also an advantage since osseointegration and bone healing can be impaired by inflammation.^54^, ^55^ However, the differences between PMSCs and MSCs from other tissues remained to be explored in the future study. Besides, whether PMSCs could be used for fabrication of cell aggregates are not clear.

Cell aggregates mimic cell‐to‐cell interactions and cell‐to‐ECM interactions that more closely reflect characteristics in native tissues. They have been widely applied in drug screening,^56^, ^57^ regenerative medicine,^19^ and tumor research.^58^ Previous studies have reported that cells could form cell aggregates using foreign materials with culture medium containing serum,^22^ hindering their large‐scale implication in the clinic. Therefore, we developed light‐controlled serum‐free method for harvesting cell aggregates in this study. Light illumination was able to induce changes in the wettability or water content of the culturing surfaces for cells.^25^, ^59^ These changes could lead to the conformational changes in or the release of the adhesive proteins or collagens, which contributed to cell detachment. In addition, electron–hole (e^−^/h^+^) pairs occur on the TiO_2_ surfaces under UV illumination,^60^ and then the potential change induced by electron accumulation would also manipulate protein release.^61^ When adopting light illumination for cell harvesting, the wavelength and light dose (depending on power density and irradiation time) must be defined first.^59^ To be specific, researchers have to consider biocompatibility and harvest efficiency since shorter‐wavelength light with a higher energy also exerts greater harm to cell viability. Generally, preliminary experiments are conducted to select a light source suitable for the cell culturing material, and to determine other parameters such as the shortest irradiation time for the most efficient harvesting. Previous studies published by our team had established a protocol based on a nanostructured TiO_2_ surface, 365 nm UV light, and irradiation duration of 30 min.^62^ The present study demonstrated that PMSCs could form cell aggregates and could be harvested through light activation. The cell aggregates were characterized by a high cell density and good viability.^63^, ^64^ Although the growth rate of isolated PMSCs would be slowed down after serum‐free culture, this strategy was only applied after cell sheet formation for the fabrication of cell aggregates, which means cell proliferation was not truly important at this point. Furthermore, they could preserve ECM and biological signals, which might facilitate the bone regeneration process.^65^, ^66^ Cell populations displaying high cadherin expression were found in the interior, whereas cells with high integrin expression were found in the exterior of the aggregates.^31^, ^67^ In this study, immunofluorescence staining demonstrated that PMSC aggregates contained a large amount of MSCs and abundant ECM. Furthermore, a live‐dead staining assay indicated that PMSC aggregates had good cell viability, which was also consistent with previous studies.^29^, ^68^ We could also infer that the serum‐free culturing and light‐induced harvesting process of PMSC aggregates in this study was safe and effective.

Cell aggregates have various advantages. First, presented stronger anti‐inflammatory effects and increased angiogenesis potential,^69^, ^70^ while immunomodulatory‐related gene expression was lower.^71^ Second, great osteogenic capacity of cell aggregates was confirmed by a number of researches^72^, ^73^, ^74^ and our in vivo studies. Cytochemical analysis, gene expression quantification, and protein expression quantification showed that MSC aggregates were associated with increased ALP activity and higher levels of expression of osteogenic markers, including osteocalcin, ALP, Runx2, collagen I, and BMPs.^74^, ^75^, ^76^ Related signaling pathways might include the Wnt/β‐catenin and BMP‐Smad pathways, as indicated by more significant upregulation of the p‐Smad1/5, p‐p38, phospho‐extracellular signal‐regulated kinase (p‐ERK), β‐catenin, and secreted frizzled‐related protein 3 (SFRP3) that was detected in the cell aggregates.^77^, ^78^

Better and faster osseointegration around titanium implants modified with PMSC aggregates was also observed in our study. Osseointegration could be influenced by implant surface characteristics, including roughness and wettability.^79^, ^80^ Since this process requires the migration, proliferation and differentiation of osteogenic cells, physical or chemical surface modification might not be effective enough to promote osseointegration.^81^ Previous studies have reported enhanced osseointegration with various cell sheet‐modified implants^62^, ^82^ and ECM sheet‐modified implants.^83^ In this study, we managed to fabricate PMSC aggregates‐implant complexes that successfully enhanced osseointegration.

Therefore, PMSC aggregates could be novel biomaterials for bone regeneration. Recently, cell aggregates have been used to deliver drugs^84^ and genes.^85^ Previous studies have succeeded in promoting bone regeneration with genetically modified dissociated cells and cell sheets.^86^ In the future, PMSC aggregates may serve as a potential drug and gene delivery vehicle to enhance their osteogenic capacity. Further in vivo studies are needed to compare osteogenic potential of PMSCs with other cell sources.

## 4 CONCLUSIONS

This study reported rat PMSCs as a new resource of MSCs with easy isolation and rapid wound healing and formed aggregates for bone regeneration. PMSC aggregates could be fabricated effectively by the light‐controlled method in scaffold‐free and serum‐free conditions. The transplantation of PMSC aggregates with no artificial scaffold successfully promoted implant osseointegration and bone defect healing. Further studies should be conducted to clarify the exact mechanism for easy formation of aggregates. Thus, these findings suggest that PMSCs could be novel cell resources for scaffold‐free bone regeneration.

## 5 MATERIALS AND METHODS

### 5.1 Isolation and culture of PMSCs, GMSCs, BMSCs, and AMSCs

In this study, all animal experiments were approved by the Institutional Animal Care and Use Committee of Zhejiang University (Hangzhou, China). Three‐week‐old male Sprague–Dawley (SD) rats were used for the isolation of PMSCs, GMSCs, BMSCs, and AMSCs. To obtain PMSCs and GMSCs, the hard palatal tissues and gingival epithelium were separated, snipped, and then cultured in basal medium (α‐MEM [Gibco, USA] supplemented with 10% FBS [Gibco, USA], 0.272 g/L l‐glutamine [Sigma, USA], 1% penicillin [Gibco, USA], and 1% streptomycin [Gibco, USA]). The wounds in the oral cavities of the rats were left to heal without further sutures. To obtain AMSCs, the subcutaneous adipose tissues were harvested from the inguinal region of the rats and also cultured in basal medium. The wounds created by adipose tissue harvesting were carefully sutured. The healing processes of PMSCs and AMSCs harvesting sites were closely observed and recorded from the second day after surgery until the tenth day. Both tissues were incubated in an atmosphere composed of 95% humidity and 5% CO_2_ at 37°C. After the first 24 h, the culture medium was changed and then replaced every 3 days. After 8 days, the remaining tissue debris was removed, and the adherent cells were digested and passaged. The medium was replaced every 2 days and passaging occurred every 4–5 days. BMSCs were isolated and cultured according to our previous protocols.^70^ The shapes and quantities of cells were visualized under a microscope (Zeiss, Germany).

### 5.2 Colony‐forming assay

In a 6‐cm dish, rat PMSCs (passage 1) were cultured at a density of 2 × 10^3^ cells/cm^2^ to evaluate their colony‐forming ability. After culturing for 7 days, PMSCs were fixed in 4% PFA (Beyotime, China) and subsequently stained with crystal violet (Solarbio, China) for 30 min. PMSC aggregates were observed under a phase‐contrast microscope (Zeiss, Germany).

### 5.3 Flow cytometric analysis of MSC markers

Passage 3 PMSCs were digested and suspended in ice‐cold 1x PBS at a density of 2 × 10^6^ cells/ml and then stained for 2 h on ice with the following antibodies: anti‐CD34 (ab81289, Abcam, UK), anti‐CD146 (ab75769, Abcam, UK), anti‐CD29 (ab179471, Abcam, UK), anti‐CD45 (ab10558, Abcam, UK), IgG H&L (ab6717, Abcam, UK), anti‐CD44 (ab23396, Abcam, UK), and anti‐CD90 (ab226, Abcam, UK). Flow cytometry analyses were conducted on a flow cytometer (CytoFLEX, Beckman, USA).

### 5.4 Trilineage differentiation of PMSCs

To evaluate the stemness of the PMSCs, trilineage differentiation experiments (osteogenic, adipogenic, and chondrogenic differentiation) of PMSCs were conducted. Briefly, PMSCs were cultured in osteogenic, adipogenic, and chondrogenic medium for 7 days. To evaluate osteogenic differentiation, rat PMSCs at passage 3 were seeded in 6‐well plates at a density of 1 × 10^5^ cells/cm^2^ and cultured with α‐MEM for 24 h until the cells adhered. Then, the basic medium was replaced with osteogenic culture medium (α‐MEM containing 10% FBS, 50 mg/ml ascorbic acid (Sigma‐Aldrich), 10 mmol/L β‐sodium glycerophosphate (Sigma‐Aldrich), and 0.1 mmol/L dexamethasone (Sigma‐Aldrich)). After culturing for 21 days, the PMSCs were fixed in 4% PFA for 15 min, and then stained with 2% alizarin red solution (ScienCell, USA) for 30 min. To assess adipogenic differentiation, rat PMSCs at passage 3 were seeded in 6‐well plates at a density of 2 × 10^4^ cells/cm^2^ and cultured with α‐MEM until the cells reached 100% confluency. Then, the basic medium was replaced with adipogenic medium A supplemented with 10% FBS, 0.1% dexamethasone, 0.2% insulin, 0.1% rosiglitazone, and 0.1% isobutylmethylxanthine (IBMX; Cyagen Biosciences, USA). After incubation for 3 days, medium A was replaced with medium B supplemented with 10% FBS and 0.2% insulin (Cyagen Biosciences, USA) for 24 h. After incubation in adipogenic medium for 21 days, PMSCs were fixed in 4% PFA for 30 min, and then stained with Oil Red O (Cyagen Biosciences, USA) for 30 min. To evaluate chondrogenic differentiation, rat PMSCs at passage 3 were seeded in 15 ml centrifuge tubes at a density of 4 × 10^5^ cells/ml and cultured with chondrogenic culture medium (Cyagen Biosciences, USA) for 28 days. Then, PMSCs were fixed in 4% PFA for 15 min, and stained with alcian blue (Cyagen Biosciences, USA) for 30 min. Samples were observed using a microscope (Zeiss, Germany). Each sample was evaluated in triplicate.

To quantify the expression levels of osteogenic, adipogenic, and chondrogenic genes, reverse transcription and quantitative polymerase chain reaction (RT‐qPCR) assays were conducted. Rat PMSCs were seeded in 6‐well plates at a density of 1 × 10^5^ cells/cm^2^ with osteogenic, adipogenic, and chondrogenic media. After 7 days of incubation, total RNA was purified using TRIzol (Invitrogen, Carlsbad, CA). Then, reverse transcription to cDNA was immediately performed with the PrimeScript RT Reagent Kit (TAKARA, China). The whole reaction was measured on an ABI ViiA7 system (Applied Biosystems, CA) using specific primers and a SYBR Green Kit (TAKARA, China). The primers for the targeted genes were as follows: *BMP2*, 5′‐ACAAACGAGAAAAGCGTCAAGC‐3′ (forward) and 5′‐CCCACATCACTGAAGTCCACATA‐3′ (reverse); *ALP*, 5′‐TGGTACTCGGACAATGAGATGC‐3′ (forward) and 5′‐GCTCTTCCAAATGCTGATGAGGT‐3′ (reverse); *PPAR‐γ*, 5′‐CCCTTTACCACGGTTGATTTC‐3′ (forward) and 5′‐CTTCAATCGGATGGTTCTTCG‐3′ (reverse); *AP2*, 5′‐CTTGGGTCGTCATCCGGTCAG‐3′ (forward) and 5′‐CCAGGGTTATGATGCTCTTCACT‐3′ (reverse); *SOX9*, 5′‐AGGCCACCGAACAGACTCAC‐3′ (forward) and 5′‐GAAGGTCTCGATGTTGGAGATGA‐3′ (reverse); *Col2a1*, 5′‐GTGGAAGAGCGGAGACTACTGG‐3′ (forward) and 5′‐TTGGGGTAGACGCAAGACTCG‐3′ (reverse); *glyceraldehyde‐3‐phosphate dehydrogenase (GAPDH)*, 5′‐GGCACAGTCAAGGCTGAGAATC‐3′ (forward) and 5′‐ATGGTGGTGAAGACGCCAGTA‐3′ (reverse). The expression levels of the target genes were calculated after normalization to GAPDH. The assays were repeated three times.

### 5.5 Cell scratch assay

Rat PMSCs, GMSCs, BMSCs, and AMSCs at passage 3 were seeded in 12‐well plates at a density of 1 × 10^5^ cells/cm^2^ for cell culture, and the culture medium was changed every 2 days. After the cell monolayer formed, a micropipette tip (200 μl) was used to gently scratch the center of the culture dishes. Then, detached cells were removed by PBS washing and the culture medium was replaced with FBS‐free α‐MEM to reduce the contribution of cell proliferation. The samples were observed at the same position under a light microscope (Zeiss, Germany) after 0, 24, 48, and 72 h. For PMSC sheets, the samples were imaged under microscope after 0, 12, 18, and 24 h. The images of 0 and 24 h were analyzed quantitatively by Image J (National Institute of Health, Bethesda, MD). Wound areas were calculated by tracing the cell‐free areas and the migration rates were expressed as the percentage of area reduction of wound closure after 24 h. The assays were repeated three times.

### 5.6 Osteogenic induction of four kinds of cells

Rat PMSCs, GMSCs, BMSCs, and AMSCs at passage 3 were seeded in 6‐well plates at a density of 1 × 10^5^ cells/cm^2^ and cultured with α‐MEM for 24 h until the cells adhered. Then, the basic medium was replaced with osteogenic culture medium. After culturing for 7 days, total RNA was purified, and reverse transcription and RT‐qPCR assays were conducted as described in 2.4. The primers for the targeted genes were as follows: *BMP2*, 5′‐ACAAACGAGAAAAGCGTCAAGC‐3′ (forward) and 5′‐CCCACATCACTGAAGTCCACATA‐3′ (reverse); *β‐catenin*, 5′‐TGGTGAAAATGCTTGGGTCG‐3′(forward) and 5′‐TCTGAAGGCAGTCTGTCGTAATAG‐3′(reverse); *LRP5*, 5′‐CTGCGATGCTGTCTGTCTCC‐3′ (forward) and 5′‐ AGCACAGTCGGGGAAGGAA‐3′ (reverse); *runt‐related transcription factor‐2 (Runx2)*, 5′‐CAGTATGAGAGTAGGTGTCCCGC‐3′ (forward) and 5′‐AAGAGOGGTAAGACTGGTCATAGG‐3′ (reverse); *GAPDH*, 5′‐GGCACAGTCAAGGCTGAGAATC‐3′ (forward) and 5′‐ATGGTGGTGAAGACGCCAGTA‐3′ (reverse). The expression levels of the target genes were calculated after normalization to GAPDH. The assays were repeated three times.

### 5.7 Inflammatory cytokines release under inflammatory environment

Rat PMSCs, GMSCs, BMSCs, and AMSCs at passage 3 were seeded in 6‐well plates at a density of 1 × 10^5^ cells/cm^2^ and cultured with α‐MEM for 24 h until the cells adhered. Then, the basic medium was replaced with medium containing TNF‐α (10602‐HNAE, Sinobiological, China) of 50 ng/ml. After 3 days of incubation, total RNA was purified, reverse transcription to cDNA and RT‐qPCR assays were conducted. The primers for the targeted genes were as follows: *IL‐1β*, 5′‐GAACAACAAAAATGCCTCGTGC‐3′ (forward) and 5′‐GACAAACCGCTTTTCCATCTTCT‐3′ (reverse); *IL‐6*, 5′‐TGGAGTTCCGTTTCTACCTGG‐3′(forward) and 5′‐GGTCTTGGTCCTTAGCCACTCC‐3′(reverse); *IL‐10*, 5′‐ACTTTAAGGGTTACTTGGGTTGC‐3′ (forward) and 5′‐ATCATTCTTCACCTGCTCCACTG‐3′ (reverse); *iNOS*, 5′‐ CACTGTGGCTGTGGTCACCTATC‐3′ (forward) and 5′‐ ACTGACACTCCGCACAAAGCAG‐3′ (reverse); *TGFβ*, 5′‐CGCAACAACGCAATCTATGAC‐3′ (forward) and 5′‐ACCAAGGTAACGCCAGGAAT‐3′ (reverse); *IFNγ*, 5′‐GGCAAAAGGACGGTAACACG‐3′ (forward) and 5′‐TTCACCTCGAACTTGGCGAT‐3′ (reverse); *GAPDH*, 5′‐GGCACAGTCAAGGCTGAGAATC‐3′ (forward) and 5′‐ATGGTGGTGAAGACGCCAGTA‐3′ (reverse). The expression levels of the target genes were calculated after normalization to GAPDH. The assays were repeated three times.

### 5.8 Cell proliferation

PMSCs at passage 3 were seeded in 24‐well plates at a density of 3 × 10^4^ cells/well and cultured with α‐MEM with 10% FBS for 4 h until the cells adhered. For the serum‐free culture group, the culture medium was changed from α‐MEM to serum‐free medium (Biological Industries). For the control group, PMSCs were cultured without changing the medium. Cell growth rates were evaluated using Alamar Blue cell viability reagent (Invitrogen, USA) at 1, 3, and 5 days. PMSCs in control group and serum‐free culture group were incubated with 10% Alamar Blue α‐MEM and 10% Alamar Blue serum‐free medium for 4 h, respectively. Subsequently, the optical density was measured at 540/590 nm using SpectraMax microplate reader (Spectra M2, Molecular Devices, USA). Culture medium supplemented with 10% Alamar Blue was used as a negative control.

### 5.9 Cell aggregate harvest

A total of 3 × 10^4^ cells were seeded in 12‐well plates. After 5 days of culturing, PMSC sheets were harvested through irradiation with light for 30 min. To avoid any interference by heat, a cold 365 nm UV light was used in this study. The transmittance power was 1.4 mW/cm^2^. The total energy was 2520 mJ/cm^2^ (<safe energy 7500 mJ/cm^2^)^87^ after illumination for 30 min. Subsequently, to promote cell aggregate formation, the culture medium was changed from α‐MEM to serum‐free medium. The PMSC sheets self‐assembled into PMSC aggregates after culture in serum‐free medium on a TiO_2_ nanodot platform for 24 h. Finally, PMSC aggregates were harvested through 365 nm UV light irradiation for 30 min.

### 5.10 Readhesion assay

A total of 1 × 10^5^ cells were seeded in 12‐well plates and cultured with α‐MEM. After 5 days of culturing, PMSC sheets were harvested through irradiation with 365 nm light for 30 min and reseeded in a new 12‐well plate. Subsequently, the culture medium was changed every 2 days. Extreme care was taken to avoid movement or floating of the sheets. After 1, 2, 3, and 4 days, adhesion of the PMSC sheets to the plates and the cell growth around the sheets were recorded using a phase‐contrast microscope (Zeiss, Germany).

### 5.11 Live‐dead staining of PMSC sheets and PMSC aggregates

To evaluate the cell viability of the PMSC sheets and PMSC aggregates, a live‐dead staining assay was performed. Briefly, PMSC sheets and PMSC aggregates were stained with calcium for 30 min and PI for 10 min at 37°C before and after illumination with 365 nm light. Cell morphology was recorded using an inverted fluorescence microscope (IX81, Olympus, Japan). Cell sheets were immersed in 4% PFA for 15 min as a negative control.

### 5.12 Immunofluorescence of PMSC sheets and PMSC aggregates

The cell sheets and cell aggregates were harvested for immunofluorescence to observe their composition and structure. They were incubated with antibodies (fibronectin and CD90) for 15 h and phalloidin for 2 h. Cell morphology was observed using an inverted fluorescence microscope (IX81, Olympus, Japan).

### 5.13 SEM

The fabricated PMSC aggregates were fixed in 2.5% glutaraldehyde for 15 h. Subsequently, PMSC aggregates were dehydrated in a graded series of ethanol solutions (30%, 50%, 70%, 80%, 90%, 95%, and 100%) for 15 min each; then, the aggregates were air‐dried and observed by SEM (SU‐8010, Hitachi, Japan). TiO_2_ nanodot platforms were assessed by SEM and EDS.

### 5.14 Bone regeneration evaluation in a tibial implant model

The in vivo osteogenic capability of PMSC aggregates around the titanium implants with a diameter of 2.2 mm and a length of 6 mm was evaluated by using 24 SD rats (3‐month‐old males; Zhejiang Academy of Medical Sciences Animal Center, Zhejiang, China). All rats were randomly divided into two groups (12 rats per group): PMSC aggregates‐implant complexes and blank SLA implants. After general anesthesia via intraperitoneal injection of 1% pentobarbital, implant cavities were prepared using 2.2 mm diameter drills, and the implants were inserted into the bilateral tibias 5 mm under the rat knee joints. To prevent the rats from infection, penicillin was injected daily after the surgery for 3 days. At 4 and 8 weeks after surgery, the tibias were dissected and then fixed in 4% PFA at room temperature for 48 h. For immunohistochemistry analysis, the tibias underwent dehydration, embedding, and paraffin sectioning. An anti‐BMP2 primary antibody (ab214821, Abcam, UK), anti‐Runx2 primary antibody (ab92336, Abcam, UK), and a goat anti‐rabbit secondary antibody (gb23303, Servicebio, China) were used for immunohistochemical staining. For hard tissue sectioning analysis, the tibias were dehydrated in increasing grades of ethanol (70%–100%) and immersed in a 1:1 mixture of 100% ethanol and Technovit 7200VLC (Heraeus Kulzer, Wehrheim/Ts, Germany), and subsequently changed to pure Technovit 7200VLC, which was later used to embed the samples. Approximately 40 μm sections were made by Leica SP1600 (Leica, Germany), stained by Toluidine blue, and imaged under microscope (Zeiss, Germany). The BIC and BV/TV were calculated using the software program Image‐Pro Plus (version 6.0; Media Cybernetics, Rockville, MD). In micro‐CT analysis, the values of BV/TV, Tb.N, Tb.Th, and Tb.Sp were calculated.

### 5.15 Bone regeneration evaluation in a tibial defect model

A rat tibial defect model was used to evaluate the osteogenic potential of PMSC aggregates in vivo. Briefly, a total of 18 rats were anesthetized with 1% pentobarbital. A round defect with a diameter of 2.2‐mm was made 2 mm below the metaphysis of the tibia. Two cell aggregates were randomly placed into the defect of the right tibia or left tibia. The control group had no tibial defects. After healing for 4 and 8 weeks, the tibias were harvested for micro‐CT analysis. The parameters of bone BV/TV, Tb.N, Tb.Th, and Tb.Sp were evaluated.

### 5.16 Statistical analysis

Statistical analysis was conducted using two‐tailed unpaired Student's *t*‐test to compare two groups or by one‐way ANOVA with Tukey–Kramer post hoc test to compare three groups. *p* <.05 was considered to be significant.

## AUTHOR CONTRIBUTIONS

**Zhiwei Jiang:** Conceptualization (equal); investigation (equal); methodology (equal). **Na Li:** Investigation (equal); writing – original draft (equal); writing – review and editing (equal). **Qin Shao:** Investigation (equal); writing – original draft (equal). **Danji Zhu:** Investigation (equal); writing – original draft (equal). **Yuting Feng:** Investigation (equal); writing – original draft (equal). **Yang Wang:** Writing – original draft (supporting). **Mengjia Yu:** Writing – original draft (supporting). **Lingfei Ren:** Writing – review and editing (equal). **Qianming Chen:** Writing – review and editing (equal). **Guoli Yang:** Supervision (equal).

## CONFLICT OF INTEREST

The authors declare no potential conflict of interest.

## Data Availability

The data that support the findings of this study are available from the corresponding author upon reasonable request.
